# The effects of mouse strain and age on a model of unilateral cervical contusion spinal cord injury

**DOI:** 10.1371/journal.pone.0234245

**Published:** 2020-06-15

**Authors:** Rebecca A. Nishi, Anna Badner, Mitra J. Hooshmand, Dana A. Creasman, Hongli Liu, Aileen J. Anderson

**Affiliations:** 1 Sue & Bill Gross Stem Cell Center, University of California-Irvine, Irvine, California, United States of America; 2 Institute for Memory Impairments & Neurological Disorder, University of California-Irvine, Irvine, California, United States of America; 3 Anatomy & Neurobiology, University of California-Irvine, Irvine, California, United States of America; 4 Physical & Medical Rehabilitation, University of California-Irvine, Irvine, California, United States of America; UCSF Weill Institute for Neurosciences, UNITED STATES

## Abstract

There are approximately 1.2 million people currently living with spinal cord injury (SCI), with a majority of cases at the cervical level and half involving incomplete injuries. Yet, as most preclinical research has been focused on bilateral thoracic models, there remains a disconnect between bench and bedside that limits translational success. Here, we profile a clinically relevant model of unilateral cervical contusion injury in the mouse (30kD with 0, 2, 5, or 10 second dwell time). We demonstrate sustained behavioral deficits in performance on grip strength, cylinder reaching, horizontal ladderbeam and CatWalk automated gait analysis tasks. Beyond highlighting reliable parameters for injury assessment, we also explored the effect of mouse strain and age on injury outcome, including evaluation of constitutively immunodeficient mice relevant for neurotransplantation and cellular therapy testing. Comparison of C57Bl/6 and immunodeficient Rag2gamma(c)-/- as well as Agouti SCIDxRag2Gamma(c)-/- hybrid mouse strains revealed fine differences in post-injury ipsilateral grip strength as well as total number of rearings on the cylinder task. Differences in post-SCI contralateral forepaw duty cycle and regularity index as measured by CatWalk gait analysis between the two immunodeficient strains were also observed. Further, assessment of young (3–4 months old) and aging (16–17 months old) Rag2gamma(c)-/- mice identified age-related pre-injury differences in strength and rearing that were largely masked following cervical contusion injury; observations that may help interpret previous results in aged rodents as well as human clinical trials. Collectively, the work provides useful insight for experimental design and analysis of future pre-clinical studies in a translational unilateral cervical contusion injury model.

## Introduction

As many as 1.2 million people are currently living with spinal cord injury (SCI), with a majority of cases involving the cervical level. Further, about half of all SCI cases involve incomplete injuries, in which the clinical effects are unilateral or do not affect both sides of the body equally [1]. Despite this reality, most preclinical research has been focused on bilateral thoracic models, highlighting the disconnect between bench and bedside, and potentially limiting translational success.

Given the anatomical and physiological differences between the cervical and thoracic spinal cord, there is growing evidence of unique pathophysiology that may affect the efficacy of therapeutic strategies [2]. Specifically, the cervical spinal cord has a larger diameter, is more vascularized, and has more gray than white matter area. There is also evidence for level-dependent variations in the immune response, where higher-level injuries may be less prone to chronic autoimmunity [3] and have a unique temporal systemic cytokine profile [4]. As a result, the translational importance of cervical injuries has been a driving force in the development of novel models.

There have been previous descriptions of unilateral cervical contusion SCI in rodents [5–8], demonstrating benefits in animal health and long-term survival, as well as the potential to assess contralateral plasticity. However, only one of these studies was in mice [6], which offer advantages for genetic knock-in and knock-out models. This study used 15 and 30 second impactor dwell times and revealed both functional impairment and histological damage contralateral to the injury [6]. Development and validation of a model with more focused and sustained ipsilateral deficits would enable more refined analysis for assessment of candidate therapies, especially for those involving stem cell transplantation, by allowing assessment of contralateral versus ipsilateral neuronal contributions to recovery. Here we describe a model with sustained ipsilateral functional deficits on multiple locomotor tasks, comparing 0, 2, 5, and 10s dwell times using the standard IH impactor device. We show that injury dwell time is useful for modulation of injury severity, allowing the model to be adjusted according to treatment strategy. Specifically, the capacity to tune dwell time could avoid ceiling and/or floor effects in recovery plateau after SCI, enabling better detection of therapeutic efficacy. We also report that a number of histological endpoints, including GFAP and lesion volume, are correlated with behavioral deficits, supporting the applicability of this model. Most importantly, based on earlier demonstrations of strain variation in responses to thoracic contusion SCI [9–13], we report data from three strains of mice, C57Bl/6 as the most commonly used background, and Rag2gamma(c)-/- as well as Agouti SCIDxRag2Gamma hybrid, as key strains for cell transplantation studies [14]. Finally, with evidence that histological damage and locomotor recovery are age-dependent, we describe the characteristics of injury in young (3–4 months old) versus aging (16–17 months old) animals. Taken together, this validation of the unilateral cervical spinal cord injury mouse model, including a novel assessment of the effects of strain and age on recovery [15], is a valuable tool for the field and for the development of further relevant preclinical studies.

## Materials and methods

**Fig 1:**
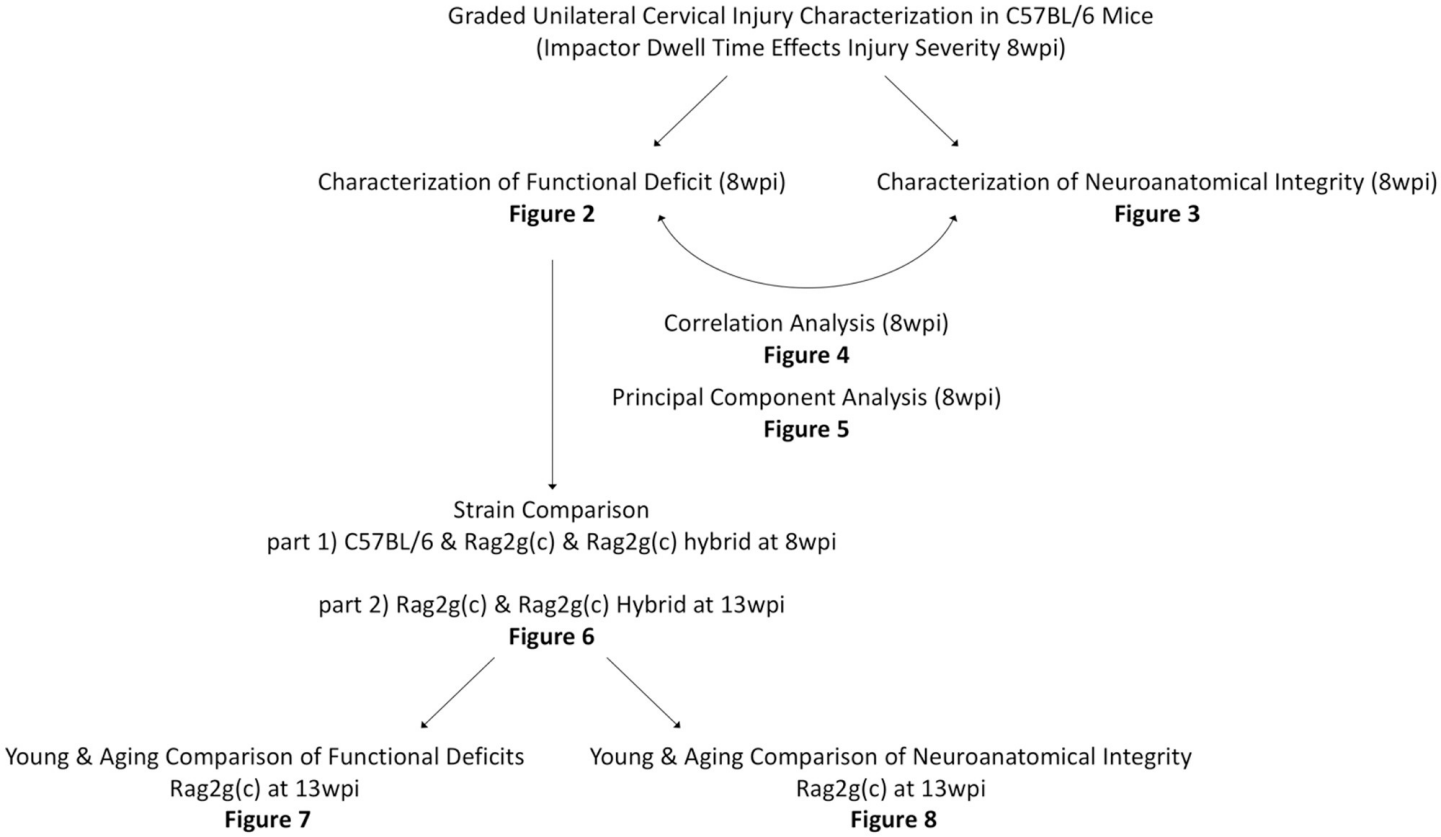
Experimental approach overview. The study design and corresponding figures are illustrated via flow chart, highlighting where data has been pooled between multiple figures.

All in vivo experiments were performed with the approval of the Institutional Animal Care and Use Committee at the University of California, Irvine. The combined experimental overview is shown in Fig 1.

### SCI surgery

8-week old female C57Bl/6 mice were divided into five injury groups (N = 12 per group) of varying severity, in which an IH Impactor (Precision Systems and Instrumentation) was used with a force of 30kD. Injury severity was increased by varying the dwell time within the IH Impactor settings (0, 2, 5, or 10 seconds) to create injuries of varying severity. It is important to note that the dwell time parameter is in addition to the spinal cord contact time, which reflects the time it takes for impactor to reverse direction. The contact time for the 0s dwell time is 4.45ms ± 0.53ms. The uninjured control group received a laminectomy alone. Under 2% Isoflurane anesthesia, mice received a laminectomy at the C5 vertebral level and the lateral vertebral processes of C4 and C6 were cleared of muscle to allow for stabilization of the vertebral column using 0.8mm tip forceps attached to the clamping platform. The impactor tip (1.0mm diameter) was consistently positioned on the right side of the spinal cord, between the midline of the spinal cord, and the lateral edge of the C5 vertebrae, to create a unilateral injury. The muscle was sutured with 5–0 chromic gut, and skin closed with 7 mm wound clips. After surgery, mice were allowed to recover overnight in cages with Alpha-Dri bedding (Newco Distributors Inc.) placed on water-jacketed heating pads at 37°C. Post-op care consisted of lactated ringers (50 ml/kg s.c.) once a day for 5 days, Baytril (2.5mg/kg, s.c.) for 2 weeks, Buprenorphine (0.01mg/kg s.c.) immediately after injury and every 12 hours for 2 days thereafter as well as manual bladder expression twice a day until mice recovered some bladder expression, then once a day for the duration of the study. All surgeries were done by personnel previously trained to perform consistent laminectomies and injuries, as validated by IH device feedback and monitoring of behavioral outcome.

For mouse strain evaluation, the 8-week old female C57Bl/6 were compared with Rag2gamma(c)-/- (Taconic) as well as Agouti SCIDxRag2Gamma hybrid (StemCells Inc) mice using a C5 vertebral unilateral contusion with a force of 30kd and 5s dwell time (N = 10–12 per group). The same parameters were used to compare the effects of age, where young (15–17 weeks) or aged (65–68 weeks) Rag2gamma(c)-/- mice underwent the aforementioned C5 vertebral unilateral contusion with a force of 30kd and 5s dwell time (N = 12–14 per group). Due to the immunodeficient nature of the Rag2gamma(c)-/- strain, additional antibiotics were administered daily throughout the 13-week duration of the study (rotating every 2 weeks the use of Baytril, Ciprofloxacin and Ampicillin, 2.5mg/kg dose for all drugs).

### Neurobehavioral tasks

All in vivo work included random allocation into study groups. All experimental data collection and analysis was completed by individuals blinded to the study groups. Further, all neurobehavioral tasks were preformed pre-injury and all animals were pre-trained on ladderbeam, CatWalk and grip strength tasks to prevent exploratory behavior.

### Grip strength

Grip strength of each forepaw was measured independently using a Dunnett-style grip strength meter [16]. Briefly, mice were supported by their trunk, positioned to grasp the metal bar and pulled back gently until they lost grip. The force of the grip strength was recorded as an average of 5 trials per animal and results were reported separately for left (contralateral) and right (ipsilateral) forepaw.

### Cylinder task

Left (contralateral) and right (ipsilateral) forelimb asymmetry was assessed using the cylinder task. Mice were placed in a glass beaker for 5 minutes, with 2 trained observers on opposite sides. The number of placements of either or both paws on the sides of the beaker were tallied and results were reported as percent of total placements for the right (ipsilateral) paw or for both paws. The total number of rearings during the testing period was also compared.

### Horizontal ladderbeam

Animals were evaluated for stepping errors when walking across a horizontal ladder with 50 rungs analyzed [17]. The task employed 4 mm diameter rungs spaced 12 mm apart. A video camera running under the ladder captured videos which were analyzed frame by frame to quantify the number of errors in three separate runs per animal. Stepping errors include missing the rung, stepping on the rung with the dorsal surface of the paw, or slipping off of the rung after placing with the plantar surface of the paw. Successful steps include stepping squarely on the rung with the plantar surface of the paw. Results were reported as the average number of errors in the left (contralateral) and right (ipsilateral) forepaws.

### Catwalk gait analysis

The Catwalk XT 9.1 gait analysis system (Noldus) is used to analyze a wide variety of gait parameters in rodents [18]. We analyzed data from 3 separate runs per animal and show the results for the commonly applied parameters following SCI, such as % step pattern, regularity index, max forepaw print area and duty cycle. Results were reported separately for left (contralateral) and right (ipsilateral) forepaw when relevant.

### Histology

At 8 weeks (cervical contusion profile) and 13 weeks (age-comparison) post-injury, mice were injected with a lethal dose of pentobarbitol followed by cardiac perfusion with 1% PBS, followed by 4% paraformaldehyde (PFA) in 1% PBS (pH 7.4) and the spinal cord dissected as previously described [19]. Spinal cord dissection took place at the injury segment, defined by the C4-C6 roots (model characterization) and C1-T2 roots (for the strain and age comparison), and samples were quickly frozen with cooled isopentane (-80°C). Sectioning took place at 30um via cryostat with Cryo-Jane tape transfer system and the slides stored at -20 °C. For immunohistochemistry, the slides were dried, heated for antigen retrieval (2.5 hours), rinsed with 1× Tris buffer and blocked (blocking solution; 0.1% Triton X and 2% donkey serum in 1× Tris buffer) for 1 hour at room temperature. Primary antibodies listed in Table 1 were diluted in blocking solution and left overnight at room temperature. After three washes (15 minutes), secondary antibodies were applied as necessary.

**Table 1:** 

Primary	Vendor	Catalog #	Dilution	Biotinylated Secondary	Company	Cat#	Dilution
MBP	Chemicon	MAB386	1:1500	Donkey anti- Rat	Jackson	712-066-153	1:500
NeuN	Millipore	ABN78	1:500	Donkey anti- Rabbit	Jackson	711-066-152	1:500
GFAP*	Dako	Z0334	1:3000	Donkey anti- Rabbit	Jackson	711-066-152	1:500

* GFAP antibody was titrated to selectively stain the dense glial scar and not individual astrocytes.

### Stereology

An Olympus BX51 microscope with motorized stage and StereoInvestigator were used for unbiased estimation of total cell numbers (Optical Fractionator probe for NeuN and MBP cell counts) and lesion volume (Cavalieri probe for GFAP negative and positive area of staining). Uniform random sampling of the tissue was performed according to standard stereological principles, where sampling parameters (i.e. grid size and counting frame size) were empirically determined to arrive at low coefficients of error (CE). CE is defined as the standard error of the mean of repeated estimates divided by the mean and used as a measure of the accuracy of the stereological procedure. The probe grid size and counting frame size were empirically determined to yield average cumulative error values <0.1. For the Cavalieri probe, the section evaluation interval was 6 with a distance of 180μm between sections. Cell counts were performed with a counting frame of 50x50μm.

### Statistics

Statistics were performed using GraphPad Prism (version 6.0). Comparison of behavioral results between groups at a single timepoint, histological lesion comparisons were analyzed for statistical significance, using one-way ANOVA, with Tukey multiple comparisons post-hoc, or t-tests where appropriate. Repeated measured ANOVA was applied for behavioral data over time, while rostrocaudal evaluation of histological data was analyzed for statistical significance using two-way ANOVA, with Tukey multiple comparisons post-hoc. The results for behavioral parameters Correlation results between histology and behavioral tasks were evaluated with parametric Pearson analysis (r and p-values reported).

Principal component Analysis (PCA) was performed using the “prcomp” function in R via RStudio. PCA is a feature extraction tool, in which an orthogonal transformation is applied to a set of input variables in order to identify a set of factors, termed principal components, that account for the variability in the dataset. Generally, only the first 1–3 PCs are needed to explain the majority of the variance, allowing the other PCs to be discarded. Further, variables that correlate will have high “loadings” (contributions) to the same PC. In this way, a data set with a large number of variables can be reduced to a smaller number of variables. Retention of PCs was determined by the “Kaiser Rule” (eigenvalue > 1), in which a PC is found to account for more variance than a single variable from the original dataset. Retained PCs were used to score each injury group, the resulting mean scores plotted, and one-way ANOVA with post-Hoc HSD tests were used to test for significant differences between individual groups.

### Exclusions

Animal exclusions were determined according to pre-hoc criteria by an investigator that was blinded to the experimental groups. In the C57Bl6 mouse cervical injury profile, N = 12 per group was planned for the study, with the exception of the most severe injury group (10s dwell) which had N = 14. Two animals were excluded from the all final data due to surgical complications (one from 2s dwell group, and one from 5s dwell group). Two additional animals were excluded from catwalk analyses as data outliers (Grubbs Test). In comparison of strain (C57Bl/6 with Rag2gamma(c)-/- mice) and age, a total of 2 animals died during the course of the study with no identified cause other than age-related mortality.

## Results

### There are sustained behavioral deficits following a unilateral cervical contusion SCI

**Fig 2:**
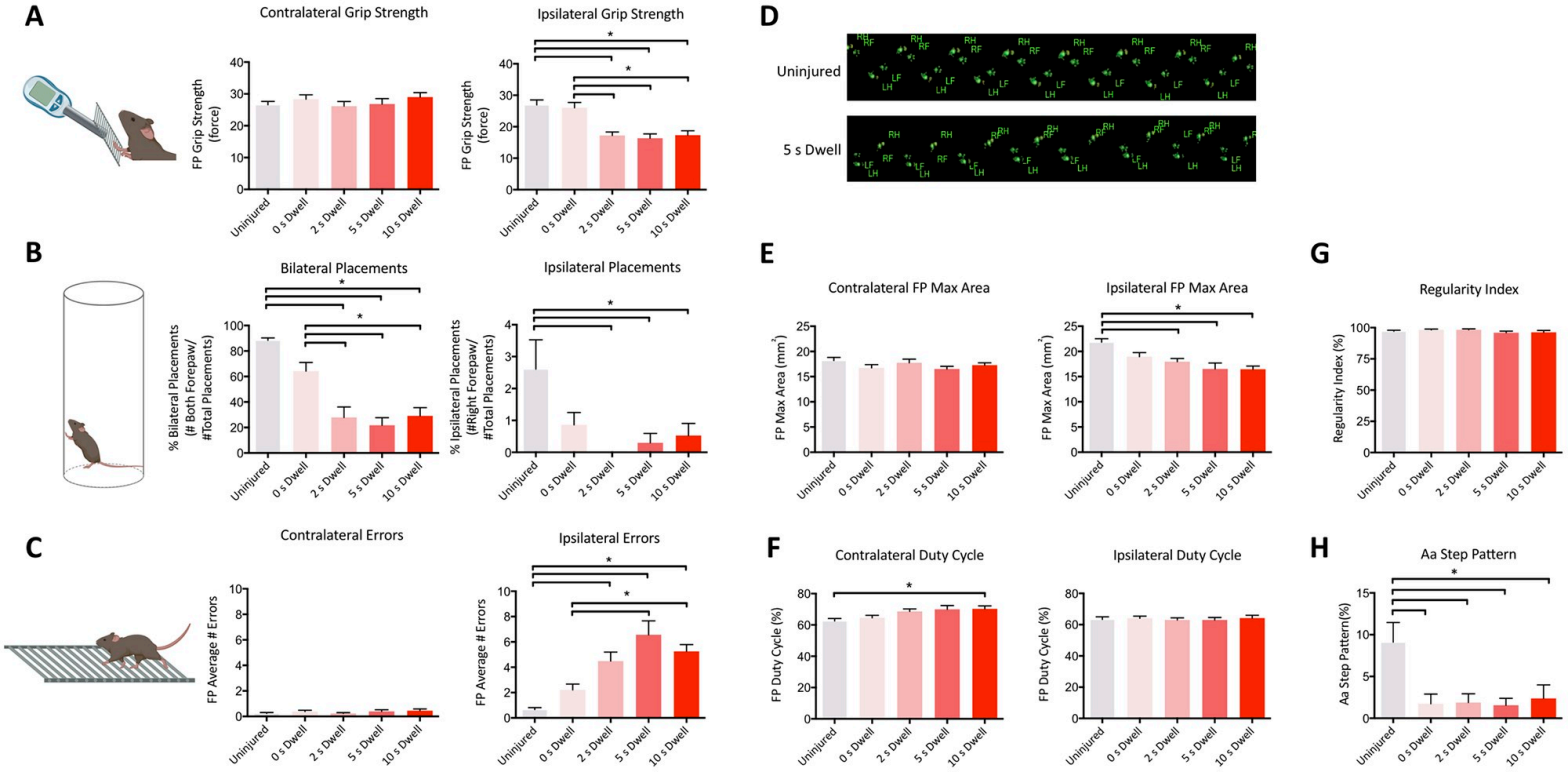
Varying impactor dwell time in the unilateral cervical contusion injury in mice (C57BL/6) led to sustained graded functional deficits as measured by forepaw grip strength (A), the cylinder test (B), horizontal LadderBeam (C), as well as CatWalk gait analysis (D-H) at 8-weeks post-SCI. Representative CatWalk gait print are shown in (D). Contralateral and ipsilateral results are shown for all relevant parameters (*n* = 12, 11, 11, 10, 14 for U, 0, 2, 5, 10 groups). Data are expressed as mean ± SEM. One-Way ANOVA, with Tukey multiple comparisons; *, p ≤ .05.

C57Bl/6 mice underwent a right-sided unilateral contusion (30kd force) at the C5 vertebral level with varying the dwell time (uninjured, 0 sec dwell, 2 sec dwell, 5 sec dwell, 10 sec dwell) of the impactor probe on the exposed spinal cord. Injury effects in grip strength, cylinder task, horizontal ladderbeam and CatWalk were sustained at the 8-week assessment (Fig 2).

Ipsilateral grip strength testing revealed a sustained impairment (Fig 2A), in which uninjured as well as injured mice with 0s dwell time could grip with a significantly stronger force (F(4,55) = 12.73, p<0.05, One-Way ANOVA, Tukey post-hoc) than those with a dwell time. There were no detectable grip strength differences between the uninjured and 0s dwell time animals or in contralateral forelimb grip strength at 8 weeks post-SCI.

The cylinder paw placement task revealed a severe impairment of the ipsilateral forepaw (Fig 2B), in which mice did not place using the ipsilateral forepaw alone. All injured animals were able to use both forepaws in unison in the cylinder, however, placement with both forelimbs was significantly different between uninjured and injured groups as well as between the injured group with no dwell time and injured groups with a dwell time (F(4,55) = 21.28, p<0.05, 1-Way ANOVA, Tukey post-hoc). Similar to the grip strength results, there were no detectable differences between the uninjured and 0s dwell time animals in bilateral or ipsilateral placements (F(4,55) = 4.12, p<0.05, One-Way ANOVA, Tukey post-hoc).

Stepping errors on the horizontal ladderbeam task also showed a persistent injury in ipsilateral forelimb function (Fig 2C), with significant differences between uninjured and injured groups with a dwell time, as well as between the injured group with 0s dwell time and injured groups with 5 and 10s dwell time (F(4,52) = 14.23, p<0.05, One-Way ANOVA, Tukey post-hoc). The contralateral forelimb paw had no difference in stepping errors.

Overall changes in gait were assessed via CatWalk parameters (Fig 2D–2H), where significant deficits in the ipsilateral forepaw function were detected in max print area (F(4,53) = 8.26, p<0.05 One-way ANOVA, Tukey post-hoc). Interestingly, the contralateral forepaw duty cycle (Fig 2F) was significantly increased in the most severe injury group (F(4,53) = 3.68, p<0.05, 1-way ANOVA, Tukey post-hoc) compared to the uninjured control group, suggesting some compensatory capacity of the contralateral side following a unilateral injury. Further, although there was no change in Regularity Index (Fig 2G), which is a broad measure of coordination, there was a significant decrease in % Aa (paw sequence RF-RH-LF-LH) step pattern (Fig 2H) of all injured groups compared to uninjured controls (F(4,53) = 4.27, p<0.05, 1-way ANOVA, Tukey post-hoc). Gait stepping patterns describe the paw placement sequence, highlighting rhythmic alternations, and have been previously described in cats with anterolateral spinal cord lesions [20]. As changes in step pattern may mask gait regularity index values, significant differences between groups are informative. The % of Aa step pattern (Fig 2H) was the only measure sufficiently sensitive to distinguish between uninjured and 0s dwell time animals.

Lastly, it is also important to note that behavioral deficits were likely unaffected by either thermal hypersensitivity or mechanical allodynia, as there were no significant differences were observed in ipsilateral or contralateral Hargreaves latency (S1 Fig). Similarly, while there was a significant difference in Von Frey ipsilateral withdrawal at 4 weeks between uninjured and injured groups, this difference was not sustained at 6 weeks, and is thus unlikely to have affected functional assessments at 8 weeks post-SCI (Fig 2).

In general, increasing the dwell time of the impactor probe led to greater injury severity as measured by ipsilateral grip strength, bilateral placements on the cylinder task, ipsilateral horizontal ladderbeam errors and CatWalk gait parameters. Importantly, most neurobehavioral tasks were unable to discriminate function between uninjured and 0s dwell time at the 8-week timepoint post-injury, apart from % Aa stepping pattern during locomotion. Further, neither task was able to identify differences between 2s and 10s dwell time, suggesting a limit in detection sensitivity. While we only report several key parameters, there are others that may highlight improvements following therapeutic intervention and should be reviewed on a case-by-case basis.

### Changes in neuroanatomical integrity following a unilateral cervical contusion SCI

**Fig 3:**
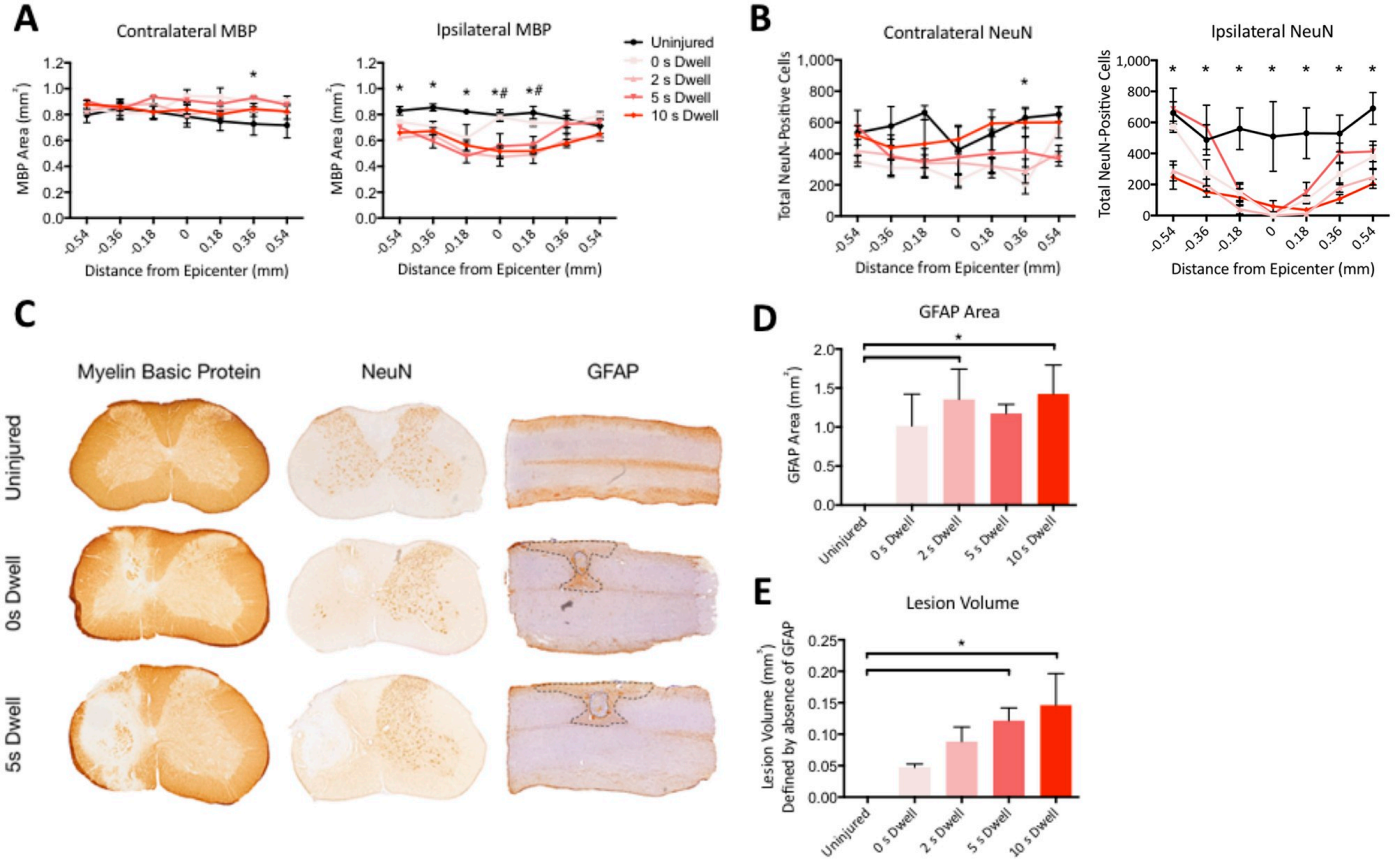
Varying impactor dwell time in the unilateral cervical contusion injury in mice (C57BL/6) led to graded changes in neuroanatomical integrity at 8-weeks post-SCI. Significant differences were detected in ipsilateral (A) myelin basic protein (MBP) area and (B) NeuN-positive cells, with representative coronal sections at the epicenter shown for uninjured as well as 0 and 5 second dwell time (C). Data are expressed as mean ± SEM (*n* = 4 per group, coronal sections). 2-Way ANOVA, with Tukey multiple comparisons between groups at each timepoint (*Uninjured vs. Injured Groups, # Injured with no dwell time vs. Injured with 2, 5, or 10s dwell time). Significant differences were also detected in GFAP area (D) and lesion volume (E) defined by absence of GFAP staining. Data are expressed as mean ± SEM (*n* = 6 per group, sagittal sections). One-Way ANOVA, with Tukey multiple comparisons; *, p ≤ .05.

Histological analysis was performed at 8 weeks following injury to evaluate the effect of dwell time on neuroanatomical integrity. White matter sparing (MBP) and neuronal loss (NeuN) were assessed via stereological analysis of sections rostral and caudal to the injury epicenter (Fig 3A–3C). Changes in white matter (MBP) were modest on the contralateral side, with only a significant difference between the 5s dwell time and the uninjured cord 0.36mm from the epicenter (Fig 3A, F(4,100) = 5.19, p<0.05, Two-Way ANOVA, Tukey post-hoc). In contrast, on the ipsilateral side, there was a significant decrease in white matter at, and rostral to, the injury epicenter between the injured groups with dwell times and the uninjured group (F(4,100) = 28.85, p<0.05, Two-Way ANOVA, Tukey post-hoc). White matter changes were not significant between different injured dwell times groups (Two-way ANOVA, Tukey post-hoc).

There was minimal neuronal loss contralateral to the injury, with a significant difference detected only between the 5s dwell time and the uninjured cord 0.36mm from the epicenter (Fig 3A, p<0.05, Two-Way ANOVA, Tukey post-hoc). On the ipsilateral side of the cord, there were significantly fewer NeuN neurons in injured versus uninjured animals (Fig 3B, F(3,60) = 11.24, p<0.05, 2-way ANOVA, Tukey post-hoc). Importantly, dwell time did not significantly affect neuronal loss at the injury epicenter, but a difference between the injured groups with varying dwell times and the injured group without a dwell time was detectable rostral and caudal to the injury epicenter (Fig 3B; p<0.05, 2-way ANOVA, Tukey post-hoc). In addition to ipsilateral and contralateral myelin and neuron loss, we also analyzed the effect of injury on two parameters of lesion size (Fig 3C). There was a significant increase in GFAP area (Fig 3D) between uninjured and 2s as well as 10s dwell time (F(4,23) = 4.11, p<0.05, 1-way ANOVA, post-hoc Tukey). Similarly, there was increased GFAP-negative lesion volume (Fig 3E) in the 5s and 10s dwell time compared to the uninjured animals (F(4,23) = 5.57, p<0.05, 1-way ANOVA, Tukey post-hoc). As reported previously, we validated the strong correlation between GFAP-negative lesion volume (the region of the scar without GFAP staining) and fibronectin in S2 Fig, demonstrating that fibronectin is found within the scar tissue devoid of GFAP.

Taken together, white matter (MBP) damage, neuron (NeuN) loss and astrogliosis (GFAP) were detected predominantly ipsilateral to the cervical contusion. As with behavioral data, most differences between injured groups with varying dwell times did not reach statistical significance. Only neuron counts rostral and caudal to the injury epicenter were sensitive enough to distinguish between the injured groups with varying dwell times and the injured group without a dwell time. Above all, the reported changes in neuroanatomical integrity demonstrate that there is a relationship with functional deficits; we explored this relationship further via correlation analysis.

### Correlations between functional recovery and histological parameters

**Fig 4:**
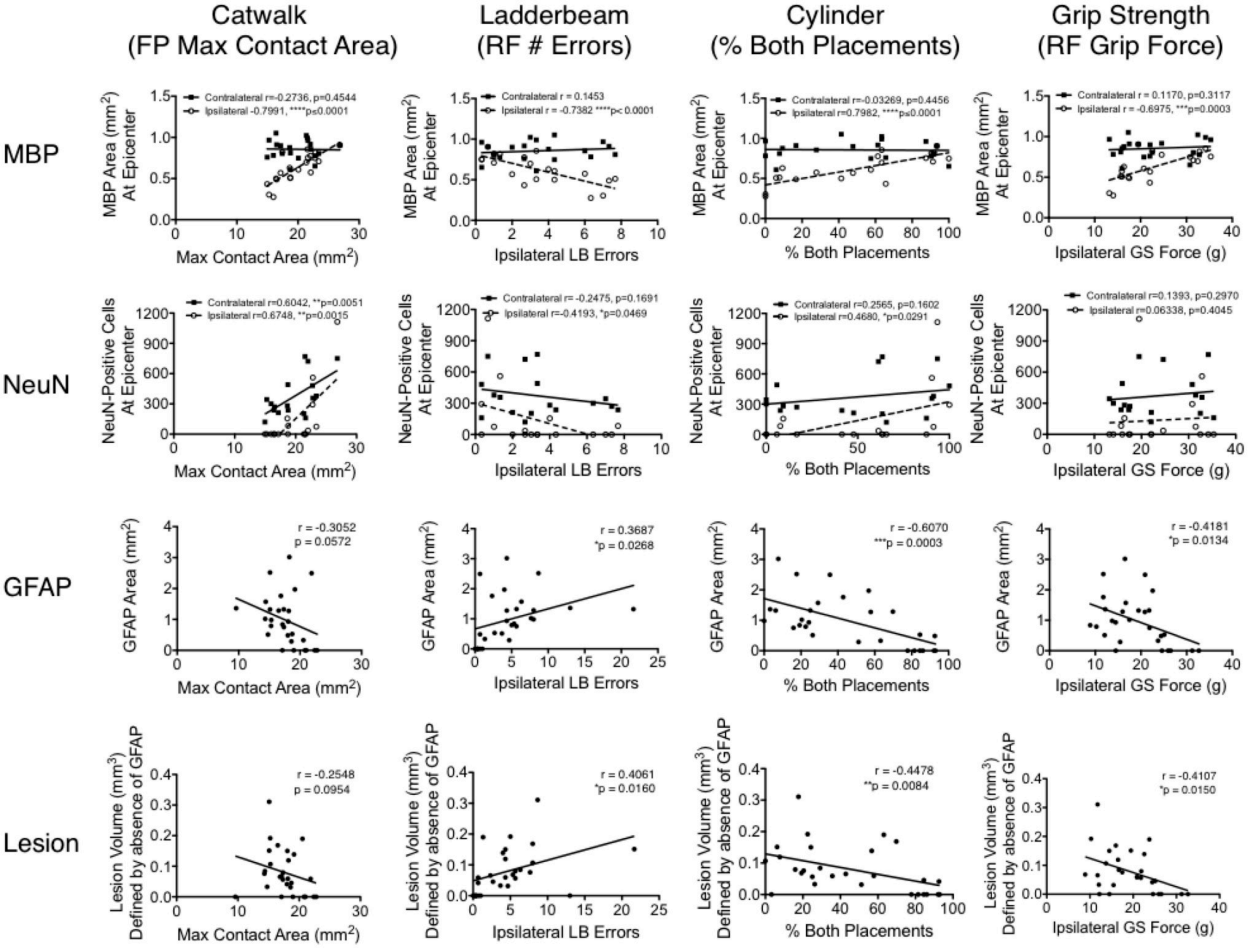
Correlation between behavioral outcomes and histological parameters following a unilateral cervical contusion injury in mice (C57BL/6) at 8 weeks post-SCI. Correlation results between MBP area, NeuN cell counts, GFAP area as well as lesion volume and behavioral tasks were evaluated with parametric Pearson analysis (r and p-values reported). These values used for correlative assessment are pooled from assessment of neuroanatomical integrity at 8-weeks post-SCI (Fig 3) and the corresponding functional outcomes at 8-weeks post-SCI (Fig 2).

With few detectable differences between injured groups of varying dwell times, correlation analysis between function and neuroanatomical integrity was predicted to reveal more subtle changes. Specifically, we compared locomotor function (including Catwalk forelimb max area, ipsilateral ladderbeam errors, percentage of both paw placements on the cylinder task and ipsilateral grip strength) with measured histological parameters (Fig 4). Importantly, there was a strong correlation (Pearson) between ipsilateral white matter sparing (MBP) and Catwalk forelimb max area (r = -0.79, p≤0.0001), ipsilateral ladderbeam errors (r = -0.74, p≤0.0001), percentage of both paw placements on the cylinder task (r = 0.79, p≤0.0001) and ipsilateral grip strength (r = -0.69, p = 0.0003).

Although there was only a weak correlation with number of neurons on the ipsilateral side and Catwalk forelimb max area (r = 0.60, p = 0.0051), there was also a correlation noted with the contralateral side (r = 0.60, p = 0.0015). This was the only significant correlation between function and contralateral histology, and could be indicative of contralateral plasticity that is involved in forelimb paw placement during gait. However, as plasticity was not evaluated in this study, this should be properly addressed in future work. The number of ipsilateral NeuN-positive cells also correlated with ipsilateral ladderbeam errors (r = -0.41, p = 0.046), as well as percentage of both paw placements on the cylinder task (r = 0.46, p = 0.029).

Specific to the ipsilateral spinal cord, GFAP as well as lesion volume displayed only a weak correlation to ipsilateral ladderbeam errors (r = 0.36, p = 0.026 for GFAP; r = 0.40, p = 0.016 for lesion), percentage of both paw placements on the cylinder task (r = -0.60, p = 0.0003 for GFAP; r = -0.44, p = 0.0084 for lesion) and grip strength (r = -0.41, p = 0.013 for GFAP; r = -0.41, p = 0.015 for lesion). In summary, the strongest correlations with function were obtained from histological assessment of ipsilateral white matter sparing (MBP) and neuron (NeuN) counts. Together, these data highlight a linear relationship between behavioral outcomes and neuroanatomical integrity using the dwell time (IH impactor setting) to create injuries with varying severity.

### Multivariate analysis of injury outcomes

**Fig 5:**
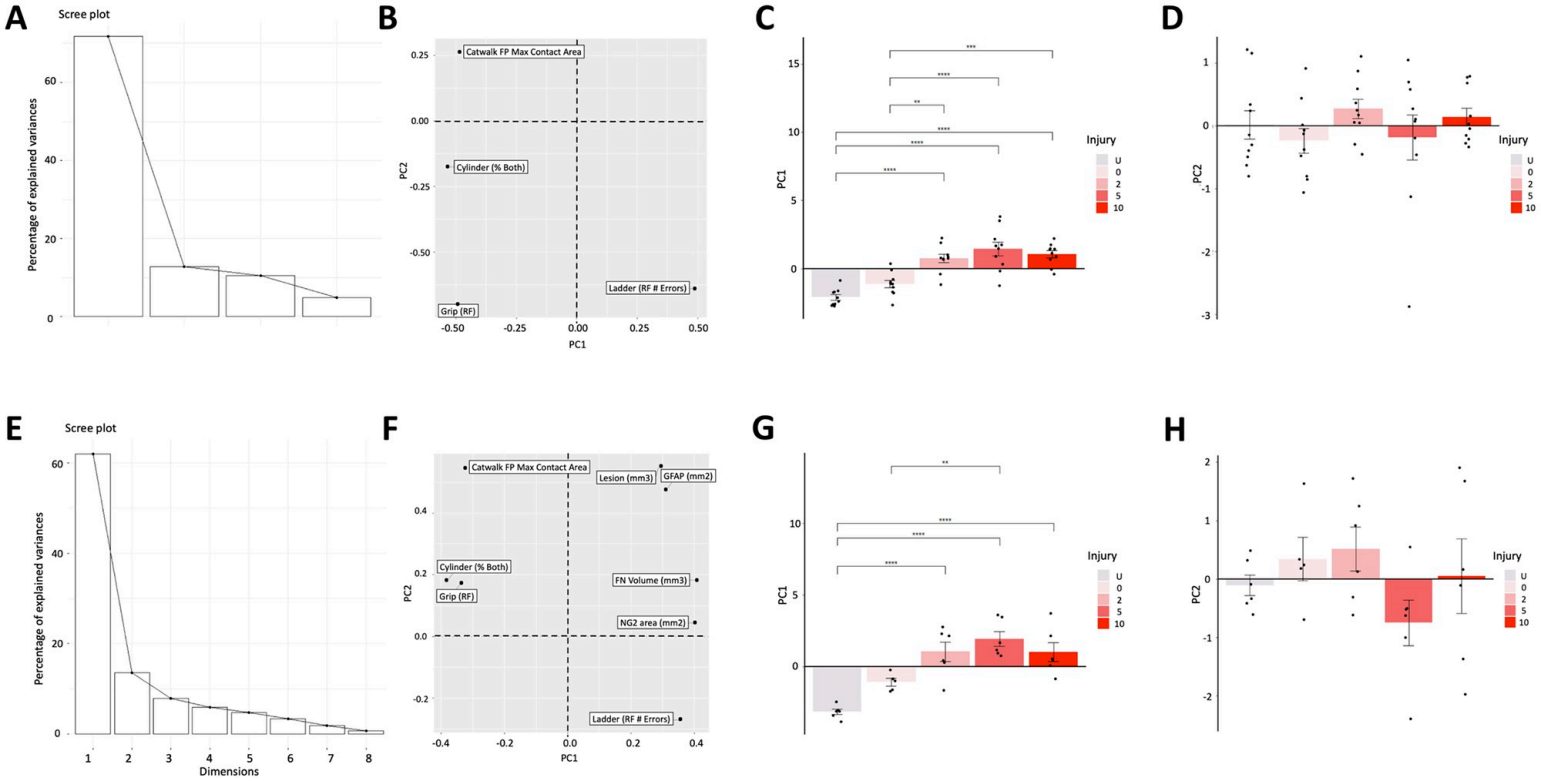
Multivariate analysis of the unilateral cervical contusion injury in mice (C57BL/6) at 8 weeks post-SCI. The scree plot (A), loading magnitude (B) and 2D plots of PC1 (C) as well as PC2 (D) on their own axes for pooled behavioral outcomes. The scree plot (E), loading magnitude (F) and 2D plots of PC1 (G) as well as PC2 (H) on their own axes for pooled behavioral combined with lesion histological outcomes. The retained PCs were used to score each subject, and the resulting mean scores for each injury group were plotted, and one-way ANOVA with post-Hoc HSD tests used to test for significant differences between individual groups; *, p ≤ .05; **, p ≤ 0.01; ***, p≤0.001; ****, p≤0.0001.

Principal component analysis (PCA) was employed to further investigate the behavioral outcomes observed to be strongly correlated with histology (Fig 5A–5D). Analysis of PC loadings for behavioral outcomes alone (Fig 5B) highlights that PC1 is similarly affected by right forepaw grip strength (Grip RF), percentage of bilateral placements on the cylinder task and CatWalk maximum forepaw contact area. Further, as errors are inversely associated with recovery, ladderbeam forepaw errors had a similar effect on PC1, but in the opposite direction. PC1 (F = 22.79, p = 2.44 x 10^−10^) accounted for ~ 72% of the variance in the dataset (Fig 5A).

Consistent with assessment of behavioral outcomes alone, analysis of PC loadings for histological lesion data in combination with behavioral outcomes (Fig 5E–5H) demonstrated that PC1 (F = 15.86, p = 1.78 x 10^−6^) was similarly affected by GFAP area, lesion as well as fibronectin volume, and ladderbeam forepaw errors. In the opposite direction, PC1 was driven by right forepaw grip strength (Grip RF), percentage of bilateral placements on the cylinder task and CatWalk maximum forepaw contact area, accounting for ~ 63% of the variance in the dataset (Fig 5B).

Overall, the results of multivariate analysis using PC1 were consistent with the dwell time groups exhibiting a more robust difference in both behavioral and histological outcome measures versus the uninjured condition than the 0s dwell time group. However, despite the suggestion of a linear correlation between behavioral outcomes and neuroanatomical measures using dwell time, no separation between the dwell time groups tested was observed.

### C57BL/6, Rag2gamma(c)-/- and SCIDxRag2Gamma hybrid strain comparison following a unilateral cervical contusion SCI

**Fig 6:**
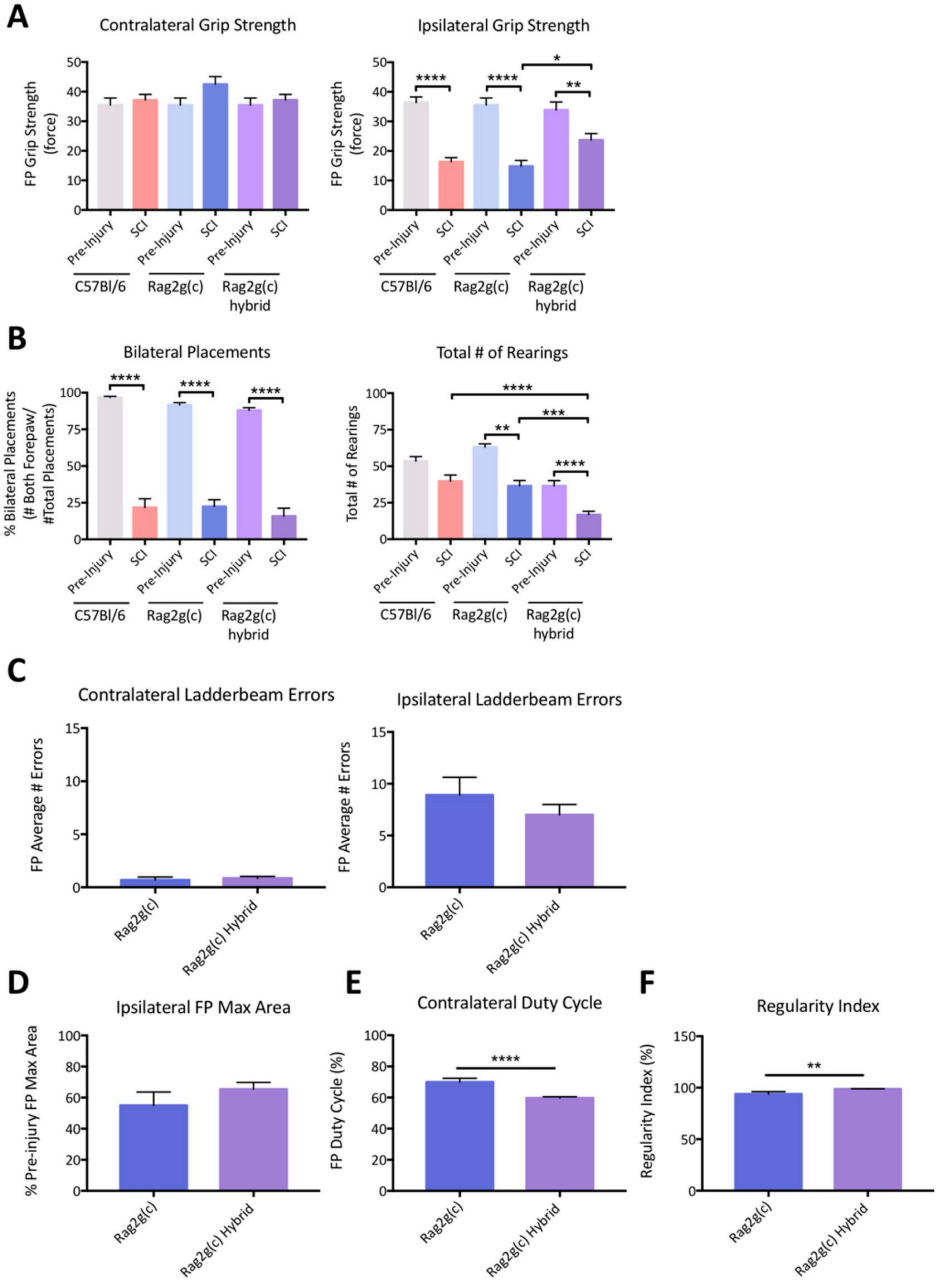
Standard C57BL/6 mice were compared at 8 weeks post-SCI with immunocompromised Rag2Gamma-/- (Rag2g(c)) as well as Agouti SCIDxRag2Gamma hybrid (Rag2g(c) hybrid) mice. While there was no difference in contralateral forepaw grip strength, the Rag2g hybrid mice had greater ipsilateral grip strength post-injury despite similar pre-injury values with Rag2g and C57BL/6 mice (A). The pre- and post-injury bilateral forepaw placements on the cylinder test were also similar between strains, although the Rag2g hybrid mice had a smaller total number of rearings pre- as well as post-injury (B). Data are expressed as mean ± SEM (*n* = 11–12 per group). One-Way ANOVA with Tukey multiple comparisons, significance is only shown for pre- and post-injury comparison between each strain as well as post-injury values between strains; *, p ≤0.05; **, p≤0.01; ***, p≤0.001; ****, p≤0.0001. At 13 weeks post-injury, average contralateral and ipsilateral forepaw errors on the horizontal LadderBeam were compared between Rag2g and Rag2g hybrid mice (C). Analysis of CatWalk gait parameters at 13 weeks post-injury revealed no difference on ipsilateral forepaw max print area (D), despite changes in contralateral duty cycle (E) and regularity index (F). Data are expressed as mean ± SEM (*n* = 11–12 per group) and the unpaired t-test applied for statistical analysis.

Overall, functional deficits following a 5s dwell time (30kD) unilateral cervical contusion were similar between C57BL/6, Rag2gamma(c)-/- (Rag2g (c)) and SCIDxRag2Gamma hybrid (Rag2g (c) hybrid) mice at 8 weeks post-SCI (Fig 6A and 6B). Nevertheless, there were several fine differences (One-way ANOVA, Tukey post-hoc) that may be important, especially in the context of potential stem cell transplant studies. Specifically, while there was no significant difference between strains in contralateral grip strength, Rag2g (c) hybrid mice displayed a much greater ipsilateral grip strength post-injury (Fig 6A). Similarly, bilateral forepaw placements on the cylinder task were comparable between strains, yet Rag2g (c) hybrid mice had significantly less total number of rearings (Fig 6B). At 13 weeks post-injury, there were no significant differences in the average number of contralateral as well as ipsilateral ladderbeam errors between Rag2g (c) and Rag2g (c) hybrid mice (Fig 6C). Yet, CatWalk gait analysis revealed a significant difference (F(3,39) = 13.66, p<0.05, t-test) in forepaw duty cycle (Fig 6E) and regularity index (Fig 6F) as measured by between the two immunodeficient strains. In all, these data demonstrate that a reproducible unilateral contusion injury can be produced within multiple mouse strains, with select strain-dependant differences. Specifically, with Rag2g (c) hybrid mice less susceptible to changes in ipsilateral grip strength. Further, Rag2g (c) and Rag2g (c) hybrid mice have small but discrete differences in gait post-injury, that may be important in assessment of cell transplant efficacy. It is also important to note that the same injury parameters were applied for C57BL/6, Rag2g (c) and Rag2g (c) hybrid mice.

### Comparison of young (3–4 months) & aging (16–17 months) Rag2Gamma mice following a unilateral cervical contusion SCI

**Fig 7:**
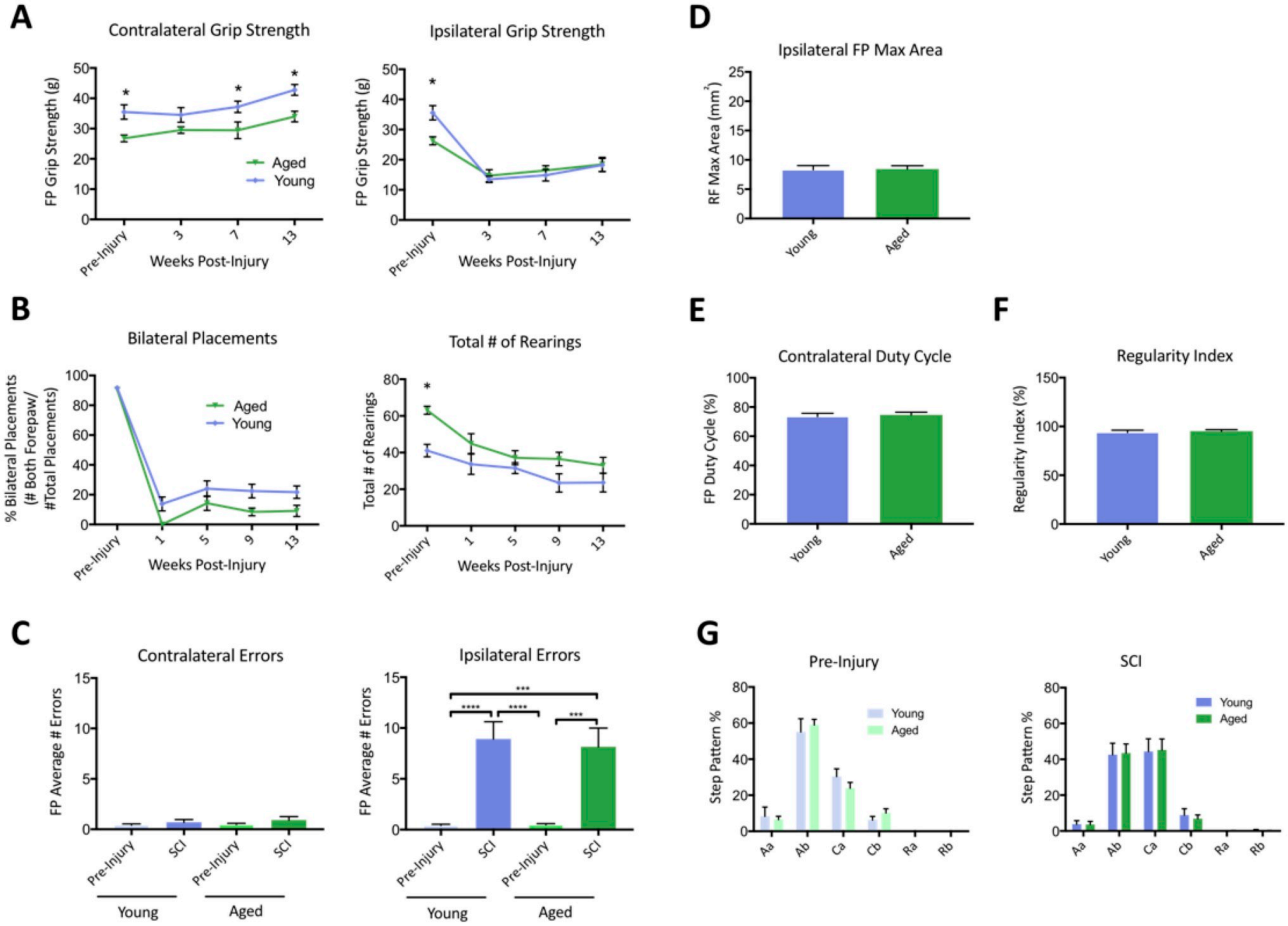
Young (3–4 months) and aging (16–17 months) immunocompromised (Rag2Gamma-/-) mice display the same functional deficits following a unilateral cervical contusion injury. The effects of Rag2Gamma mice age are shown via ipsilateral and contralateral forepaw grip strength (A), bilateral forepaw placements as well as total number of rearings on the cylinder test (B) over 13 weeks post-SCI. Data are expressed as mean ± SEM. Two-Way ANOVA, with Sidak's multiple comparisons test; *, p ≤ .05. There are also no age-mediated effects on pre-injury and post-injury contralateral as well as ipsilateral forepaw errors on the horizontal LadderBeam (C) at 13 weeks post-SCI. Data are expressed as mean ± SEM (*n* = 11–12 per group). One-Way ANOVA, with Tukey multiple comparisons; ***, p≤0.001; ****, p≤0.0001. The primary CatWalk gait parameters unilateral cervical contusion injury (D-G) are also unaffected by age (two-tailed t-test). Data are expressed as mean ± SEM (*n* = 11–12 per group).

As age is thought to play a role in pathogenesis post-SCI, functional deficits and histological parameters were compared between young (3–4 months) and aging (16–17 months) Rag2Gamma mice post-SCI. In uninjured animals, there were significant (p<0.05, Two-Way ANOVA, Tukey post-hoc) age-related differences in contralateral and ipsilateral grip strength (Fig 7A). Interestingly, while ipsilateral grip strength was similarly impaired in young and aging mice post-SCI, there were modest differences preserved for the contralateral forelimb, reaching significance at weeks 7 and 13 (F(1,92) = 29.34, p<0.05, Two-Way ANOVA, Tukey post-hoc). Together, these data suggest that the compensatory increase in contralateral grip strength post-SCI is likely hindered in aging animals.

There was also a minor decrease in the percentage of placements of both paws during the cylinder task in aging mice following injury (Fig 7B), but this did not reach significance (Two-Way ANOVA, Tukey post-hoc). Nevertheless, not surprisingly, the total number of rearings during the cylinder task was significantly decreased (F(1,23) = 12.46, p<0.05, Two-Way ANOVA, Tukey post-hoc) in aging mice pre-injury. There were no differences (One-way ANOVA, Tukey post-hoc) between young and aging mice in horizontal ladderbeam contralateral and ipsilateral stepping errors (Fig 7C), CatWalk forelimb max area (Fig 7D), contralateral duty cycle (Fig 7E) regularity index (Fig 7F) and step pattern (Fig 7G) both pre- and post-injury.

**Fig 8:**
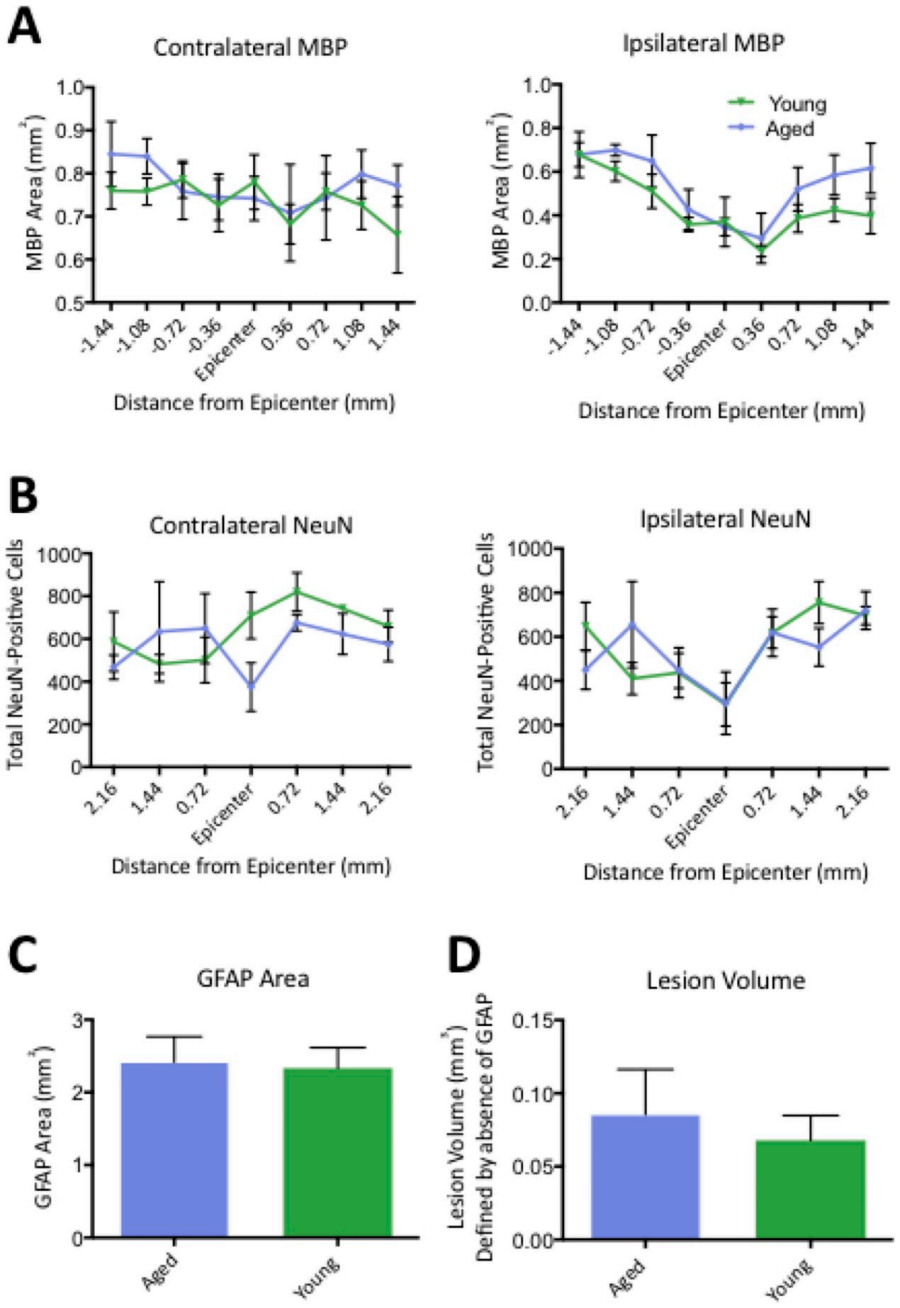
Young (3–4 months) and aging (16–17 months) immunocompromised (Rag2Gamma-/-) mice have the same changes in neuroanatomical integrity following a unilateral cervical contusion injury. The effects of Rag2Gamma-/- mice age are shown via contralateral and ipsilateral myelin basic protein (MBP) area (A) as well as contralateral and ipsilateral NeuN-positive cells counts (B) at 13 weeks post-SCI. Data are expressed as mean ± SEM (*n* = 6 per group). Repeated Measures ANOVA, with Sidak's multiple comparisons test. There are also no age-mediated effects (two-tailed t-test) on GFAP area (C) and lesion volume (D) defined by absence of GFAP staining. Data are expressed as mean ± SEM (*n* = 6 per group).

In support of the modest behavioral differences between young and aging mice, there were no effects (Two-Way ANOVA, Tukey post-hoc) in contralateral and ipsilateral white matter sparing (Fig 8A) or neuron loss (Fig 8B). There was also no difference (One-way ANOVA, Tukey post-hoc) in GFAP area (Fig 8C) or lesion volume defined by the absence of GFAP-staining (Fig 7D). Overall, this confirms that age-related effects are not a result of lesion pathogenesis in Rag2Gamma mice, demonstrating that this model can be readily applied for studies in aging.

## Discussion

In this study, we describe sustained behavioral deficit and tissue pathology following a unilateral cervical contusion spinal cord injury (SCI) in mice. Beyond highlighting reliable parameters for injury assessment via a varying dwell time paradigm, the work also explores mouse strain and age effects on injury outcome. The comparison between C57BL/6, Rag2gamma(c)-/- (Rag2g (c)) and SCIDxRag2Gamma(c)-/- hybrid (Rag2g (c) hybrid) mice strains provides evidence of fine differences in deficit post-injury, specifically in ipsilateral grip strength and total number of rearings on the cylinder task. There were also differences in forepaw duty cycle and regularity index between Rag2g (c) and Rag2g (c) hybrid mice at 13 weeks post-SCI. Altogether, this study provides useful insight for experimental design and analysis of future pre-clinical studies in two translational SCI models.

As a most clinical spinal cord injuries are incomplete and at the cervical level, a reliable research model to serve as a basis for basic research studies and testing therapeutics is critical for progress in the field [21]. Further, previous unilateral cervical models in mice have been limited to a single severity with a floor effect on many behavioral tasks [6], diminishing the potential for recovery with therapeutic intervention. In contrast, there have been more reports of the unilateral cervical model in rats [5,8,22], demonstrating sustained motor [8], sustained sensory [5], and multivariate outcome analysis [22]. Expanding on these previous studies to the development of a mouse model, this study employed IH impactor dwell time (Figs 2 and 3) to enable tailoring this model for unique research purposes. More importantly, deficits in locomotor function are correlated with various histological parameters (Fig 4), providing a useful tool. This finding is also supported by multivariate analysis (Fig 5), which detected significant differences between uninjured, 0 dwell time injuries, and 2-10ms dwell time injuries. Importantly, however, these data also suggest that increasing dwell time beyond 2ms did not result in additional separation.

Interestingly, unlike the previous unilateral cervical model in mice [6], our injury was predominantly restricted to the ipsilateral spinal cord with most correlations between function and neuroanatomical integrity being ipsilateral. There was a single strong correlation between the contralateral neuron counts (NeuN-positive cells) immunoreactivity and CatWalk functional recovery at 8 weeks. While there may have been other more discrete contralateral changes, these were not sustained over the 8-week assessment period. There were also no sustained effects on mouse allodynia (S1 Fig). Together, these unexpected findings may be indicative of potential on-going plasticity post-SCI and could also be useful for the study of contralateral neuronal contributions to recovery in preclinical studies. The role and effects of plasticity remains an important mechanistic consideration following treatment in SCI [23] and should be the focus of future work.

Despite several reports of mouse strain differences in functional recovery and tissue pathology following thoracic SCI [9–13], the role of strain has been largely unexplored in cervical injuries, especially in a unilateral model. Therefore, this study aimed to compare functional deficits between the prevalent C57BL/6 and two common strains for xenograft cell transplantation studies, Rag2g (c) and Rag2g (c) hybrid mice, which lack T, B, and NK cells, making them effective transplant hosts for human cells. Most importantly, through this comparison, we validate that the injury model can be produced multiple mouse strains, as all displayed functional deficits in ipsilateral grip strength (Fig 6A) and bilateral forepaw placements on the cylinder task (Fig 6B). However, interestingly, Rag2g (c) hybrid mice displayed greater ipsilateral grip strength post-injury compared to the two other strains. While it is unclear whether this difference could affect interpretation of treatment efficacy result, it is notable.

Similarly, young and aged mice were compared in their response to the unilateral cervical contusion injury. Previous work with thoracic models of SCI has shown aged rodents have less locomotor recovery, a larger area of pathology and more axonal demyelination compared to younger rats [24]. Further, in a thoracic model, we have previously shown that aged rats have less locomotor recovery, a larger area of pathology near the injury epicenter, increased cell death caudal to the injury epicenter and a localized increase in macrophage infiltration [15]. More specific to mice, age-effects have also been assessed in the context of secondary pathogenesis, where aged mice were found to have greater reactive oxygen species (ROS)-mediated damage and macrophage activation following a thoracic injury [25]. Albeit, this work failed to look at the effects on long-term functional recovery. Here, using Rag2g (c) mice, there were no post- cervical injury differences between young and aged mice (Figs 7 and 8). Although there were pre-injury differences in grip strength and number of rearings during the cylinder task, SCI-mediated deficits were similar in both groups. Consistent with these behavioral results, there were also no differences in histological lesion parameters (Fig 8). Importantly, as mentioned above, Rag2g (c) mice are deficient in T, B and NK cells, which are essential for transplantation studies involving human cells, but these deficits may also mask any potential age-related differences in injury pathogenesis. Relatedly, the interleukin-2 receptor subunit gamma (Il2rg), which is involved in cytokine signaling [26], is also absent in Rag2gamma mice and may further contribute to the absence of age effects on injury. Future work should explore the role of immune cells in aging and age-effects on SCI pathophysiology. Equally notable is the reality of comorbidities found in an aging population; these may confound the effect of age alone on injury [27].

Collectively, this work provided a thorough characterization of the unilateral cervical contusion injury, with sustained functional deficits detectable on multiple behavioral tasks. Although the goal was to develop a gradient of neuroanatomical and functional deficits, only ipsilateral ladderbeam error values were sensitive enough to detect the differences between different dwell times. Further, although it increasingly recognized that a single outcome is unlikely to capture the complexity of impairment post-SCI [22] and multiple assessments are necessary, multivariate analysis was also unable to significantly discriminate between the dwell time groups (Fig 5). This result is consistent with previous reports of graded SCI models [28,29], which are unable to show a consistent gradient on all neuroanatomical as well as functional outcomes. This is likely exacerbated by the complexity of the cervical spinal cord. Nonetheless, we demonstrate that the injury is mainly contained to the ipsilateral spinal cord and has no floor effects in functional recovery, which is especially important for translational preclinical studies. In addition to model characterization, this work reports on mouse strain and age effects in response to injury that will provide a solid framework for future studies.

## Supporting information

S1 Fig Varying impactor dwell time in the unilateral cervical contusion injury in mice (C57BL/6) led to changes in nociceptive sensitization as measured by hindpaw withdrawal to Von Frey filaments with a diameter of 4.08 (A) and 4.31 (B) as well as Hargreaves test (C) for thermal hyperalgesia. Contralateral and ipsilateral results are shown for all relevant parameters (*n* = 12, 11, 11, 10, 14 for U, 0, 2, 5, 10 groups). Data are expressed as mean ± SEM (Repeated Measures ANOVA, with Sidak's multiple comparisons test).
(TIF)

S2 Fig Fibronectin volume correlates with GFAP-negative lesion volume. Data are expressed as mean ± SEM (parametric Pearson analysis) with r and p-values reported.
(TIF)

S1 Method (DOCX)
