# Supplementary figures and images for: The effects of mouse strain and age on a model of unilateral cervical contusion spinal cord injury
Source: PLoS One. 2020 Jun 15;15(6):e0234245. doi: 10.1371/journal.pone.0234245 (PMC7295191; doi:10.1371/journal.pone.0234245)

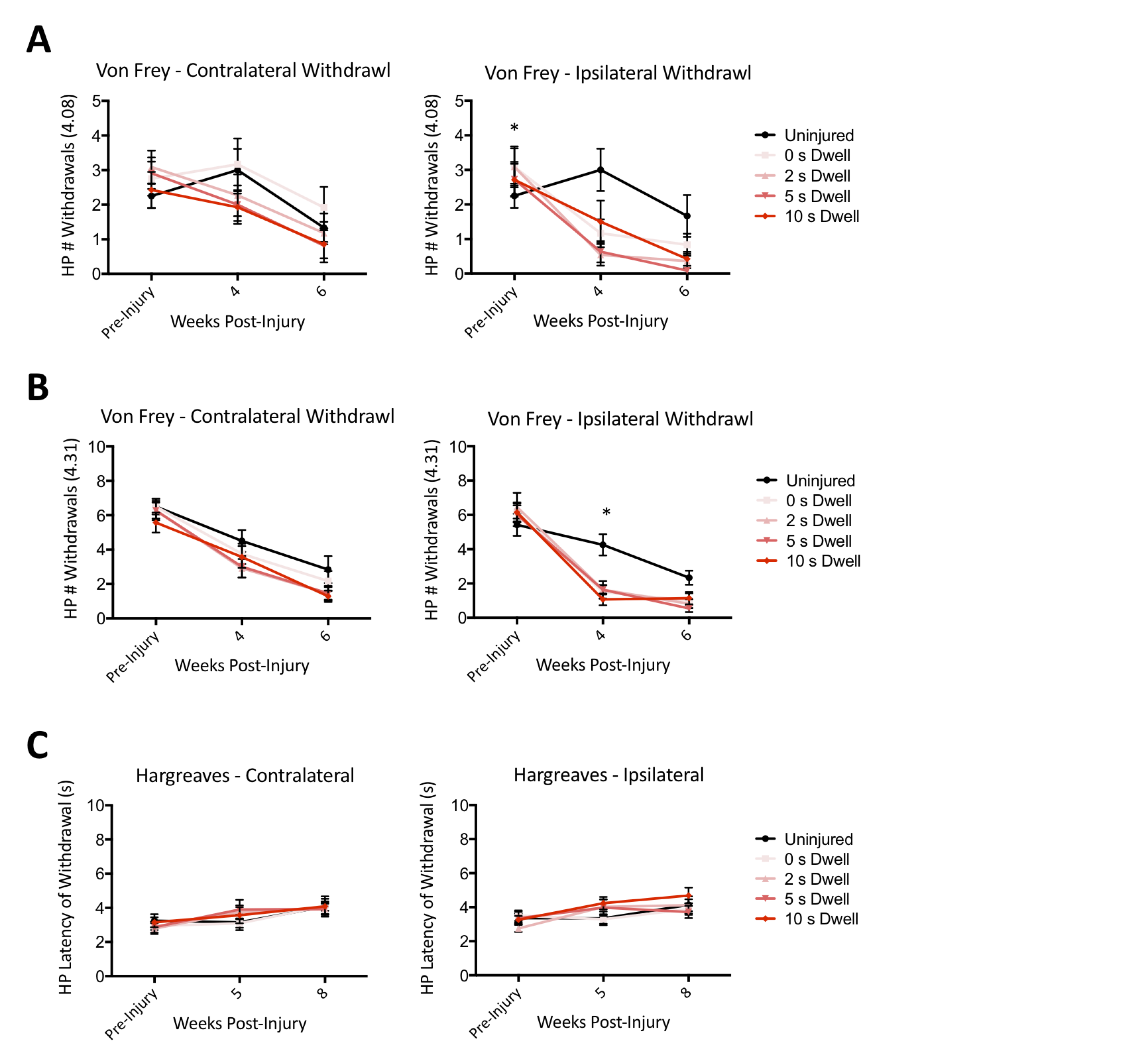

Supplement: S1 Fig — Contralateral and ipsilateral results are shown for all relevant parameters (n = 12, 11, 11, 10, 14 for U, 0, 2, 5, 10 groups). Data are expressed as mean ± SEM (Repeated Measures ANOVA, with Sidak's multiple comparisons test). (TIF) [file pone.0234245.s001.tif]

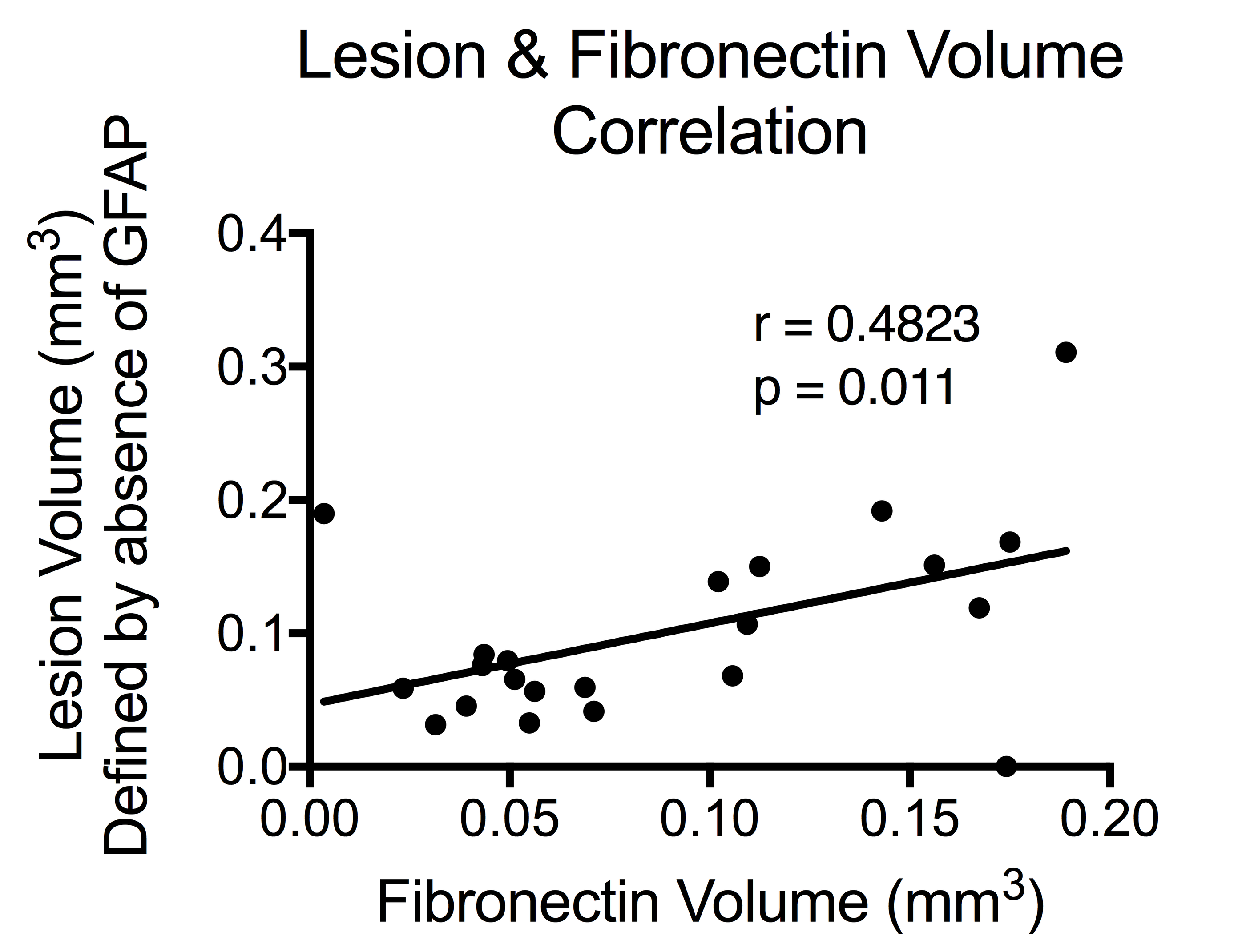

Supplement: S2 Fig — Data are expressed as mean ± SEM (parametric Pearson analysis) with r and p-values reported. (TIF) [file pone.0234245.s002.tif]
