# Supplementary material for: The effects of mouse strain and age on a model of unilateral cervical contusion spinal cord injury
Source: PLoS One. 2020 Jun 15;15(6):e0234245. doi: 10.1371/journal.pone.0234245 (PMC7295191; doi:10.1371/journal.pone.0234245)
Supplement: S1 Method — (DOCX) [file pone.0234245.s003.docx]

**Supplementary methods**

**Von Frey Test:**

Mechanical allodynia was assessed using Von Frey hair testing. Animals are placed on a mesh grid and hind paws are touched with a Von Frey hair (4.08 or 4.31 filament). Results were reported the number withdrawal for left (contralateral) and right (ipsilateral) hindpaw.

**Hargreaves Test:**

Thermal hyperalgesia was tested using the Hargreaves test. Briefly, animals were placed on a warmed glass plate and tested with a beam of 38˚C heat. The time for the hindpaw to withdraw was a measure of the sensitivity of the paw to heat. Results were reported as latency of withdrawal (seconds) for left (contralateral) and right (ipsilateral) hindpaw.

**Statistical analysis for Von Frey and Hargreaves Test:**

Statistics were performed using GraphPad Prism (version 6.0), where comparison between groups over time was analyzed for statistical significance with the repeated measures ANOVA.
